# The Reinforcement Effect of Nano-Zirconia on the Transverse Strength of Repaired Acrylic Denture Base

**DOI:** 10.1155/2016/7094056

**Published:** 2016-06-02

**Authors:** Mohammed Gad, Aws S. ArRejaie, Mohamed Saber Abdel-Halim, Ahmed Rahoma

**Affiliations:** ^1^Department of Substitutive Dental Sciences, College of Dentistry, University of Dammam, P.O. Box 1982, Dammam 31411, Saudi Arabia; ^2^Department of Biomaterial Dental Sciences, College of Dentistry, University of Dammam, P.O. Box 1982, Dammam 31411, Saudi Arabia; ^3^Department of Dental Materials, College of Dentistry, Al-Azhar University, P.O. Box 11117, Assiut, Egypt

## Abstract

*Objective.* The aim of this study was to evaluate the effect of incorporation of glass fiber, zirconia, and nano-zirconia on the transverse strength of repaired denture base.* Materials and Methods.* Eighty specimens of heat polymerized acrylic resin were prepared and randomly divided into eight groups (*n* = 10): one intact group (control) and seven repaired groups. One group was repaired with autopolymerized resin while the other six groups were repaired using autopolymerized resin reinforced with 2 wt% or 5 wt% glass fiber, zirconia, or nano-zirconia particles. A three-point bending test was used to measure the transverse strength. The results were analyzed using SPSS and repeated measure ANOVA and post hoc least significance (LSD) test (*P* ≤ 0.05).* Results.* Among repaired groups it was found that autopolymerized resin reinforced with 2 or 5 wt% nano-zirconia showed the highest transverse strength (*P* ≤ 0.05). Repairs with autopolymerized acrylic resin reinforced with 5 wt% zirconia showed the lowest transverse strength value. There was no significant difference between the groups repaired with repair resin without reinforcement, 2 wt% zirconia, and glass fiber reinforced resin.* Conclusion.* Reinforcing of repair material with nano-zirconia may significantly improve the transverse strength of some fractured denture base polymers.

## 1. Introduction

Denture fracture is a common problem in prosthodontic practice that troubles both patients and prosthodontists. Inordinate masticatory forces or denture deformation during use can result in bending forces that contribute to fatigue of the material and subsequent fracture [1]. A new denture construction increases the cost and is time consuming, so denture repair is preferred [2]. Satisfactory repair should be easy and rapid and match the original color of the denture base while maintaining the dimensional accuracy [3]. Denture repair depends on many variables including material type, material reinforcement, surface design, and surface treatment [2]. Several materials have been used to repair fractured denture bases, including autopolymerized, visible light polymerized, heat polymerized, or microwave polymerized acrylic resin [4, 5]. Most (86%) of denture base repairs are made with autopolymerized acrylic resin [6] because it is easy to manipulate and fast and can be used chair-side [7]. Unfortunately, its strength has been shown to range from 18 to 81% of intact heat polymerized denture resin [3, 8]. Many attempts have been made to overcome this shortcoming via using reinforced repair material and/or modification of repair surface design and treatment. Hanna et al. investigated the effect of 45° bevel of the repair surface on the transverse strength of the repaired denture base and found that higher values were obtained [9]. Beveling of the repair surface changed the fracture type from weak adhesive to strong cohesive fracture [10]. It is appropriate to treat the repair surface with repair monomer as it modifies the surface structure and increases its bond to repair material [11–13].

Glass fiber is one of the most common reinforcement materials and many investigations of its effect on repaired denture base have been performed. Addition of glass fiber to repair material improves the strength of a denture base repair and may decrease the occurrence of future fracture [3, 14, 15]. This may be attributed to the fact that glass fiber has a high resilience which allows the stresses to be received by them without deformation [16].

Zirconia (ZrO_2_) is a metal oxide and may be used as a reinforcement material to improve the transverse strength of denture base resin [17, 18]. Reinforcement of acrylic denture base with zirconia significantly increases its transverse strength [19]. Recently, nanotechnology invaded the prosthodontic field for medical and material enhancement purposes. The properties of the reinforced resin by nanoparticles depend on the size, shape, type, and concentration of the added particles [20]. Additions of nano-zirconia to polymethylmethacrylate (PMMA) denture base have been reported to increase the transverse strength due to its small size and homogenous distribution [21].

The disadvantage of commonly used repair materials is that they have poor strength. The current research in the field of dental materials is focused on finding the appropriate repair material with adequate strength and prolonged shelf life. Till date, the effect of nano-ZrO_2_ on repair strength has not been investigated. Therefore, this study was conducted to evaluate the reinforcement effect of different concentrations of glass fiber, zirconia, and nano-zirconia on the transverse strength of a repaired denture base. The null hypothesis was that the addition of different concentrations of zirconia or nano-zirconia will not improve the transverse strength of repaired denture base.

## 2. Materials and Methods

In accordance with ANSI/ADA specification number 12, eighty rectangular specimens of heat polymerized acrylic resin with dimensions (65 × 10 × 2.5 mm ± 0.1) were prepared using customized molds [22]. Molds were waxed up (Cavex Set Up Wax, Cavex, Netherlands) and then wax patterns were invested in type III dental stone (GC Fujirock EP, Belgium) within a flask (61B Two Flask Compress, Handler Manufacturing, USA) and then dewaxed to create the mold space. According to the manufacturer's instructions, heat polymerized acrylic resin (Major Base 20, Major Prodotti Dentari SPA, Italy) was mixed and packed in the dough stage into the mold cavity and trial closure was done and then flask was closed and kept under bench press for 30 min. Flask with acrylic resin specimens was processed for 8 hours in water bath at 74°C and then temperature was increased to 100°C for 1 hour into thermal curing unit (KaVo Elektrotechnisches Werk GmbH, D-88299, Germany). After curing, the flasks were bench cooled to room temperature prior to deflasking. The excess resin of deflasked specimens was removed with a tungsten carbide bur (HM251 FX 040 HP, Meisinger, USA), polished with acrylic polisher (HM251FX-060, Meisinger, USA), and then stored in distilled water at 37°C for 48 hours. All specimens were randomly divided into eight groups: one intact and seven repaired groups (Table 1). To create 3 mm repair gap, repair specimens were placed into the mold and numbered on both ends for reassembling. Mark was drawn at the specimen center and then at 1.5 mm distance from this mark two lines were drawn on both sides and perpendicular to the long edge of specimen. These two lines were extended on the surfaces of the mold as a guide for all specimens. At these lines the specimens were cut with low speed diamond disc (DeguDent, GmbH, REF 59903107, Dentsply, Germany) under profuse irrigation. Standardized 45° bevel joint was prepared by measuring a 2.5 mm and drawing a line parallel to the prepared edge. In the same manner, the mold sides were cut at the center measuring 8 mm from the upper surface and 3 mm from the lower surface preserving the mold base intact. Specimens were placed in the mold and cut in bevel direction by diamond disc guided by lines and mold surfaces to create a repair gap of 3 mm × 10 mm × 2.5 mm with a 45° bevel joint. Glass fiber (E-glass; length = 3 mm, Shanghai Richem International Co., Ltd., China), zirconia (99.5%, 5 um, 1314-23-4, Shanghai Richem International Co., Ltd., China), and nano-zirconia powder (99.9%, <100 nm, 1314-23-4, Shanghai Richem International Co., Ltd., China) were weighed using an electronic balance (S-234; Denver Instrument, Germany) in a concentration of 2 wt% and 5 wt% of autopolymerized acrylic resin powder (Major Repair; Major Prodotti Dentari SPA, Italy). Preweighed glass fiber, zirconia, and nano-zirconia powder were separately added to the autopolymerized acrylic resin powder and thoroughly mixed using a mortar and pestle to achieve an equal distribution of particles and uniform color. According to numbering, specimen sections were reassembled into the original mold and fixed creating 3 mm between reassembled sections. The repair surfaces were treated with the methyl methacrylate monomer for three minutes. Repair was done using the sprinkle-on monomer-polymer method and slightly overfilling the repair gap to compensate polymerization shrinkage and finishing procedures. Once the surface of the repair material lost its glaze, the molds and their contents were placed in the pressure chamber containing water at (40°C) and at pressure 30 IB/inch^2^ (pound-force per square inch) for 15 minutes. After curing, the specimens were removed from the mold, finished, polished, and then put into distilled water and incubated at 37°C for 48 hours and then tested [3, 23]. To determine transverse strength, fracture load was measured using the three-point bending test on a universal testing machine (INSTRON 8871, Servo Hydraulic system, Merlin 2 software). The specimens were placed on a 3-point flexure apparatus and the support span was 50 mm. Load was applied at the midpoint of the repaired area with crosshead speed of 5 mm/min until the specimen fractured and fracture load was recorded. The formula

(1) $$\begin{array}{c} \left(\;TS=\frac{3WL}{2{bd}^{2}}\;\right) \end{array}$$

was used to calculate the transverse strength values of each specimen, where TS is the transverse strength (in MPa), *W* is the fracture load (N), *L* is the distance between the two supports, *b* is the specimen width, and *d* is the specimen thickness [24, 25].

## 3. Statistical Analysis

Data analysis was performed by using SPSS-20.0, IBM software, Chicago (USA). The results were presented as mean and standard deviations. Repeated measure ANOVA was applied to see the statistical significance of the variables in comparison with control group and AP. Post hoc least significance (LSD) test was used to see the pairwise comparison of the variables. *P* value ≤0.05 was considered statistically significant result.

## 4. Results

The mean value and standard deviation of transverse strength are summarized in Table 2. The statistical analysis revealed that the transverse strength of the HC was the highest strength value between tested groups (Figure 2). There were statistically significant differences in transverse strength between the repaired groups 5NZR, 2NZR, 2GF, and 5ZR as compared to AP (*P* ≤ 0.05). The higher transverse strength values were in groups 5NZR, 2NZR, and 2GF, respectively. Meanwhile 5ZR showed a significant decrease in transverse strength value. There was no significant difference in transverse strength between 2ZR and 5GF with AP.

## 5. Discussion

**Figure 1:**
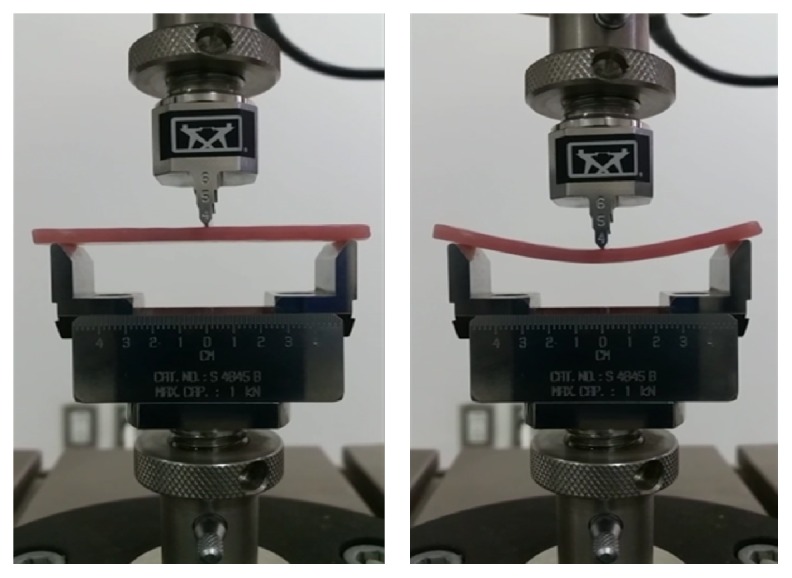
Acrylic resin specimen loaded on universal testing machine and subjected to fracture load.

**Figure 2:**
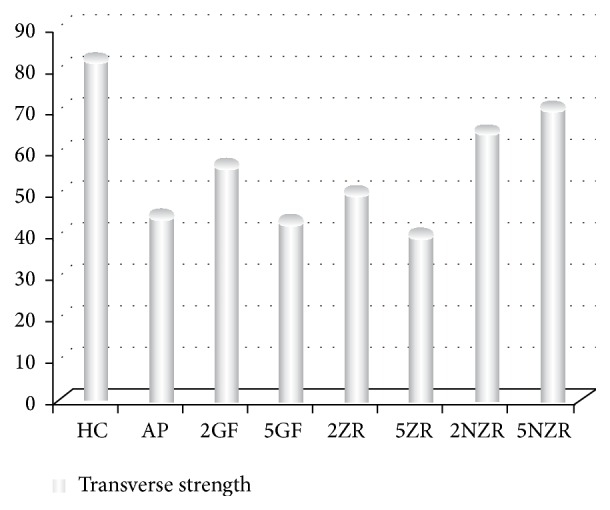
Mean value of transverse strength for all tested groups.

This* in vitro* study was carried out to evaluate the reinforcing effect of different concentrations of glass fiber, zirconia, and nano-zirconia on the transverse strength of a repaired denture base. Results revealed that the HC group had the highest transverse strength values amongst all groups, which is in agreement with the results of a previous study [26]. Some reinforced repaired specimens exhibited an increase in transverse strength compared to AP; hence, the null hypothesis was rejected. The transverse strength of AP decreased up to half the value of HC group, which is in agreement with the results of previous studies [3, 27, 28]. The decrease in transverse strength may be due to lower strength of autopolymerized acrylic resin; insufficient polymerization process; and the residual monomer retained at the repair site [29–31] (Figure 1). Glass fiber addition to repair material was found to improve the transverse strength of the repaired denture base and may be more acceptable for use because of aesthetics and ease of use [32]. Findings of the current study revealed an increase in the transverse strength of 2GF compared to AP, which is in agreement with the results of a previous study [32]. This increase may be attributed to the fact that glass fiber has a high resilience which allows the stresses to be received by them without permanent deformation [16]. 5GF showed a decrease in transverse strength, which is in agreement with a previous study [33]. This could be explained due to the high fiber content which might affect the bond strength between the repair material and the denture base [34]. The results of this study showed that the addition of 2ZR improved the transverse strength of the repaired specimens. This increase in transverse strength might be resulting from the transformation of zirconia from the tetragonal to monoclinic phase resulting in absorbing the energy of crack propagation in a process called transformation toughening. Also, in this process, expansion of ZrO_2_ crystals occurs and places the crack under a state of compressive stress and arresting the crack propagation [35]. The results of the present study showed that the transverse strength decreased in proportion to zirconia concentration. 5ZR additions resulted in a significant decrease in transverse strength compared to AP. This reduction in transverse strength may be caused by many reasons including higher filler percentage which resulted in more defects that affect the material strength; clustering of the particles within the resin; and more filler particles after reaching saturation of matrix leads to interruption in the resin matrix continuity [36, 37]. In contrast, one study reported that the transverse strength increased as zirconia content increased [19]. Results of this study showed that the transverse strength significantly increased after incorporation of 2NZR [38]. This increase in the transverse strength may be due to good distribution of the nanosize particles which enable them to enter and fill the spaces between polymeric chains resulting in increased interfacial shear strength between the nanoparticles and polymeric chains which improve the transverse strength [39]. It was also observed that the maximal transverse strength was recorded with 5NZR [21, 39], and the increase in nano-zirconia percentage increases the transverse strength, which is in agreement with a previous study [39] while being in disagreement with other studies [21, 40]. Clinical implication of the present study is that the incorporation of nano-zirconia into autopolymerized repair resin enhances the strength repaired denture base. The study design could not mimic the clinical conditions; hence this limitation affected the testing procedures and mechanical property investigated. Future research to study these materials should focus on simulation of clinical conditions with existing prosthesis and implementation of appropriate tests.

## 6. Conclusion

According to the results and limitations of this* in vitro* study, it could be concluded that nano-zirconia may be considered as a new approach for denture base repair. The repairs resulted in significantly higher transverse strength as compared to unreinforced repaired resin.

## Figures and Tables

**Table 1:** Tested groups and coding according to repair material reinforcement.

Group code	Repair material
HC	Intact heat polymerized specimens (control)
AP	Autopolymerized acrylic resin
2GF	Autopolymerized acrylic resin reinforced with 2 wt% glass fiber
5GF	Autopolymerized acrylic resin reinforced with 5 wt% glass fiber
2ZR	Autopolymerized acrylic resin reinforced with 2 wt% zirconia
5ZR	Autopolymerized acrylic resin reinforced with 5 wt% zirconia
2NZR	Autopolymerized acrylic resin reinforced with 2 wt% nano-zirconia
5NZR	Autopolymerized acrylic resin reinforced with 5 wt% nano-zirconia

**Table 2:** Mean, standard deviation (SD), and *P* values for different concentrations of glass fiber, zirconia, and nano-zirconia reinforcement.

	Mean ± SD	Versus HC	Versus AP
HC, control	83.01 ± 3.03	—	—
AP	44.85 ± 3.68	—	—
2GF	56.98 ± 2.58^*∗∗*^	0.0001	0.001
5GF	42.75 ± 2.45^*∗*^	0.0001	0.175
2ZR	50.07 ± 2.97^*∗*^	0.0001	0.064
5ZR	40.21 ± 3.31^*∗∗*^	0.0001	0.035
2NZR	65.43 ± 2.62^*∗∗*^	0.001	0.0001
5NZR	70.77 ± 2.80^*∗∗*^	0.001	0.0001

^*∗*^Statistical significance of the material with control group only at *P* ≤ 0.05.

^*∗∗*^Statistical significance of the material with control as well as AP at *P* ≤ 0.05.
